# Canine leishmaniosis caused by *Leishmania major* and *Leishmania tropica*: comparative findings and serology

**DOI:** 10.1186/s13071-017-2050-7

**Published:** 2017-03-13

**Authors:** Gad Baneth, Daniel Yasur-Landau, Matan Gilad, Yaarit Nachum-Biala

**Affiliations:** School of Veterinary Medicine, Hebrew University, P.O. Box 12, Rehovot, 76100 Israel

**Keywords:** *Leishmania major*, *Leishmania tropica*, Cutaneous leishmaniosis, Canine leishmaniosis, Israel, Co-infections

## Abstract

**Background:**

Infection and clinical disease associated with *Leishmania major* and *Leishmania tropica*, two common agents of human cutaneous leishmaniosis, have rarely been reported in dogs. This study describes dogs infected with these *Leishmania* spp. prevalent in the Middle East and North Africa, and compares the serological response of dogs infected with *Leishmania infantum*, *L. major* or *L. tropica* to whole promastigote antigen enzyme-linked immunosorbent assay (ELISA) of each species and to rK39 dipstick.

**Results:**

*Leishmania major* infection in a 5-month-old male dog was associated with alopecic and ulcerative periocular and limb skin lesions which responded to allopurinol treatment. Infection was detected by skin and blood polymerase chain reaction (PCR) and confirmed by DNA sequencing but the dog was seronegative. *Leishmania tropica* infection was detected in a 3-month-old female dog co-infected with *Babesia vogeli* and *Anaplasma platys* and with no skin lesions. PCR and DNA sequencing of the blood and parasite culture were positive for *L. tropica*. Sera from 11 dogs infected with *L. infantum*, *L. major* or *L. tropica* were reactive with all three *Leishmania* spp. antigens except for sera from a dog with *L. major* infection. No significant differences were found between reactivity of dog sera to the antigen of the infecting species, or to the other *Leishmania* spp. antigens. Sera from dogs infected with *L. infantum* and *L. tropica* were positive with the rK39 antigen kit, while dogs with *L. major* infection were seronegative.

**Conclusions:**

Skin lesions in *L. major* infected dogs from this study and previous reports (*n* = 2) were ulcerative and located on the muzzle, feet and foot pads and not associated with generalized lymphadenomegaly and splenomegaly. In previous *L. tropica* infections, skin lesions were proliferative mucocutaneous in young dogs (*n* = 2), or associated with widespread dermatitis, lymphadenomegaly and splenomegaly in older dogs with similarity to *L. infantum* infection (*n* = 2). This study suggests that ELISA serology with whole promastigote antigen is not distinctive between *L. infantum*, *L. major* and *L. tropica* canine infections and that some *L. major* infections are not seropositive. PCR with DNA sequencing should be used to discriminate between canine infections with these three species.

**Electronic supplementary material:**

The online version of this article (doi:10.1186/s13071-017-2050-7) contains supplementary material, which is available to authorized users.

## Background

*Leishmania major* and *Leishmania tropica* cause human cutaneous leishmaniosis in Asia and Africa with infringement of *L. tropica* also into Greece in southern Europe [1, 2]. In the Middle East and Israel, these two *Leishmania* spp. are common causes of human infection with wildlife mammal reservoirs, with *Phlebotomus papatasi* as the sand fly vector for *L. major*, and *Phlebotomus sergenti* and *Phlebotomus arabicus* as vectors of *L. tropica* [1, 3, 4]. Domestic dogs have been shown to suffer from clinical disease associated with infection with these two *Leishmania* spp., although *L. major* and *L. tropica* are considered rare causes of leishmaniosis in dogs in the Old World, compared to *L. infantum*, and a study from southeastern Iran has detected clinical *L. tropica* infection verified by PCR in only two of 471 (0.4%) dogs in the endemic Kerman region [5]. Likewise, descriptions of the clinical characteristics and laboratory test findings of these two canine infections are scant and there are no established protocols for their detection or knowledge on what should be expected in serological testing of these infections. The aim of this study was to describe additional clinical canine cases of *L. major* and *L. tropica* infections, compare them to previous ones [6, 7] and study the serological response to infection using antigens of three different *Leishmania* spp.

## Methods

### Dogs

Dogs diagnosed with *L. tropica* or *L. major* infection during 2015–2016 at the Hebrew University School of Veterinary Medicine (HUSVM) laboratory for vector-borne infectious diseases were included in the study. Data on history, clinical signs, hematology and serum biochemistry and specific tests for the diagnosis of leishmaniosis were collected from each case and samples were taken for parasitological detection of the disease.

### *Leishmania* PCR

*Leishmania* detection was performed by realtime PCR using primers JW11/JW12 targeting a 120 bp sequence of the *Leishmania* short fragment from the kinetoplast minicircle [8]. Additional detection and identification was carried out by high resolution melt (HRM)-PCR using primers ITS-219 F and ITS-219R to amplify a 265 bp fragment of the *Leishmania* ribosomal operon internal transcribed spacer 1 (ITS1) region and then evaluated by HRM analysis as previously described [9]. PCR was performed using the StepOnePlus real-time PCR thermal cycler (Applied Biosystems, Foster City, CA, USA) as previously described [10]. DNA samples extracted from parasite promastigote cultures of *L. infantum*, *L. tropica* and *L. major* were used as positive controls for each corresponding PCR and DNA from colony-bred dogs negative by PCR for vector-borne pathogens was used as a negative control. A non-template control (NTC) with the same reagents described above but without DNA was added to each PCR to rule out contamination.

All positive PCR products were sequenced using the BigDye Terminator v3.1 Cycle Sequencing Kit and an ABI PRISM 3100 Genetic Analyzer (Applied Biosystems, Foster City, CA, USA) at the Center for Genomic Technologies, Hebrew University of Jerusalem, Israel. DNA sequences were evaluated with the ChromasPro software version 2.1.1 (Technelysium Pty Ltd., Australia) and compared for similarity with sequences available in GenBank, using the BLAST program (http://www.ncbi.nlm.nih.gov/BLAST/).

### Parasite culture

*Leishmania* promastigotes grown in culture were prepared as crude antigen for serology as previously described [11]. The strains used for antigen production were *L. infantum* MCAN/IL/1994/LRC-L639, *L. tropica* MHOM/IL/2005/LRC-L1239 and *L. major* MHOM/TM/1973/5ASKH. The antigens were prepared for serological testing carried out in 96-well enzyme-linked immunosorbent assay (ELISA) plastic plates coated with 1.5 μg protein per well.

### Serology

Serology for the dog cases was performed for the initial diagnostic procedures using *L. infantum* antigen by ELISA as previously described [11]. Subsequently, ELISA serology was performed with three different *Leishmania* spp. antigens: *L. infantum*, *L. major* and *L. tropica* for each serum sample available from the *L. major*- and *L. tropica*-infected dogs, including also for sera from previously described canine *L. tropica* and *L. major* cases [6, 7]. In addition, sera from eight dogs with clinical *L. infantum* disease diagnosed in the HUSVM and confirmed by HRM-PCR [9] and DNA sequencing were also evaluated. All sera were tested for reactivity with each of the three *Leishmania* spp. antigens at the same time. All dog sera, tested at 1:100 dilutions, were incubated with one of the three different leishmanial antigen coated plates for 1 h at 37 °C. The plates were then washed with 0.1% Tween 20 in 50 mM phosphate-buffered saline (PBS), pH 7.2, and incubated with Protein A conjugated to horseradish peroxidase (1:10,000 dilution; Zymed Laboratories, Inc., San Francisco, CA, USA) for 1 h at 37 °C. Excess conjugate was removed by extensive washing in PBS-Tween and the plates were developed by addition of the substrate 2,29-azino-di-3-ethylbenzthiazoline sulfonate (ABTS) (Boehringer Mannheim, Mannheim, Germany). Each plate was read when the absorbance (lambda = 405 nm) of the positive canine reference serum reached a value between 1.2–1.4. Dilutions of positive and negative reference dog sera were included on each plate to monitor interassay variation.

All results were adjusted to the highest values obtained from serum from a dog infected with the same species of *Leishmania* as the plate coating antigen, e.g. for a plate coated with *L. infantum* antigen, all results were adjusted to serum from a dog infected with *L. infantum*. Each serum was run in triplicate and the final optical density (OD) value was calculated as the average of readings. OD values were adjusted for each antigen separately and eventually sera from each dog infected with a certain *Leishmania* species were compared with readings of its reactivity with other *Leishmania* spp. antigens and with sera of other dogs infected by the same species, in order to investigate if using a homologous *Leishmania* antigen is beneficial for the diagnosis of the infecting species. Serological cut-off values for each *Leishmania* sp. were calculated based on two standard deviations above the mean OD value of readings from eight control sera from seronegative and PCR-negative dogs.

Antibodies reactive with the recombinant antigen rK39 were tested using the Kalazar Detect dipstick kit (InBios International Inc., Seattle, Washington, USA) according to the manufacturer's instruction.

### Statistical analysis

All data distributed normally as tested by the Shapiro-Wilk test. A One-Way ANOVA was used to determine the mean differences between OD values obtained for serum of dogs infected with the same *Leishmania* spp. or with different *Leishmania* spp. antigens. Statistical analysis was performed using the SPSS® 21.0 software (IBM, Armonk, New York, USA).

## Results

### Clinical cases

#### Case no. 1

**Fig. 1:**
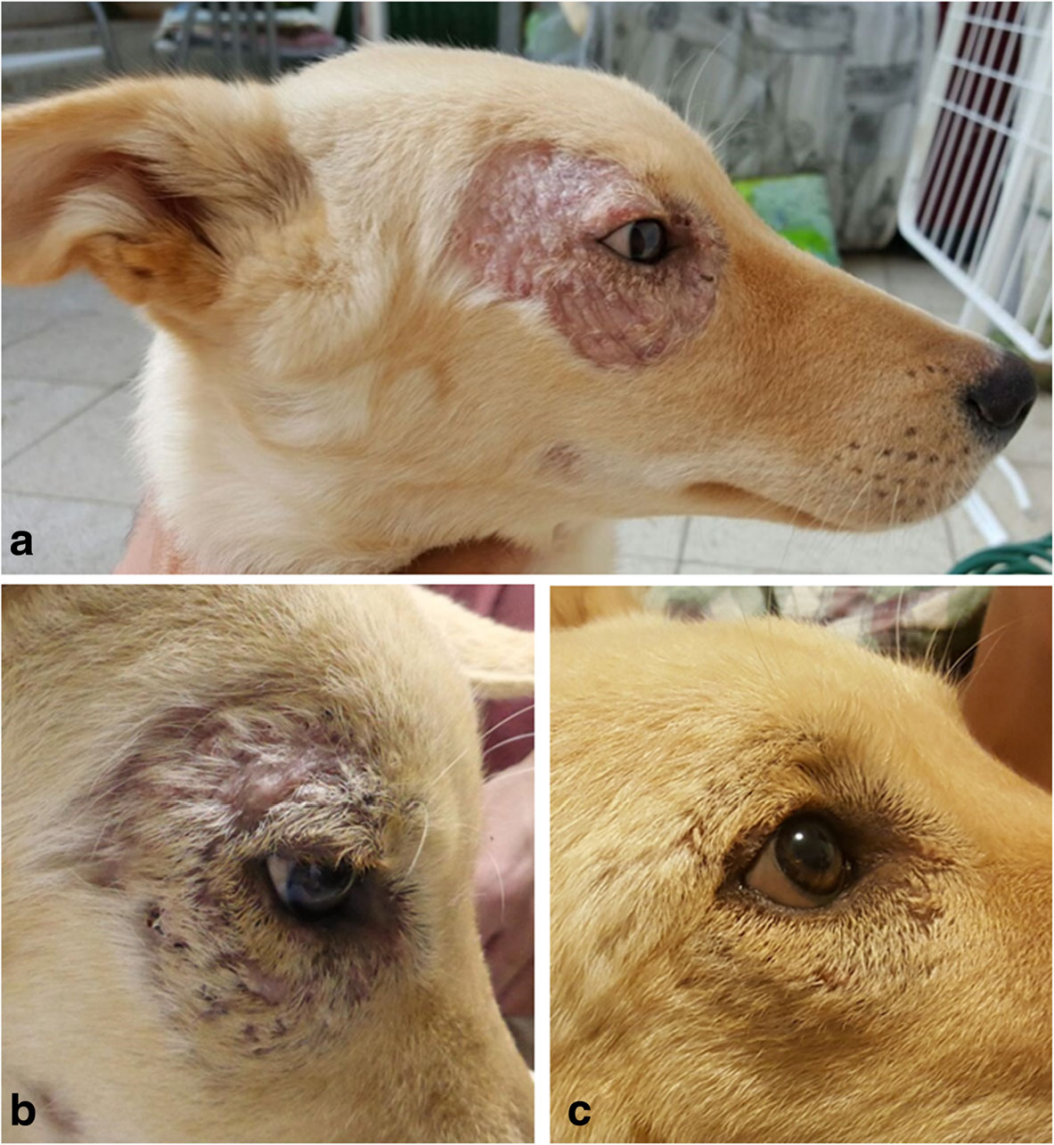
Cutaneous periocular *Leishmania major* skin lesion in dog case no. 1 with changes over time: **a** on initial diagnosis at 6 months of age; **b** at 7 months of age on the day of begining allopurinol treatment. Some healing and growth of hair can be observed even before treatment; **c** 7 weeks after begining treatment

**Fig. 2:**
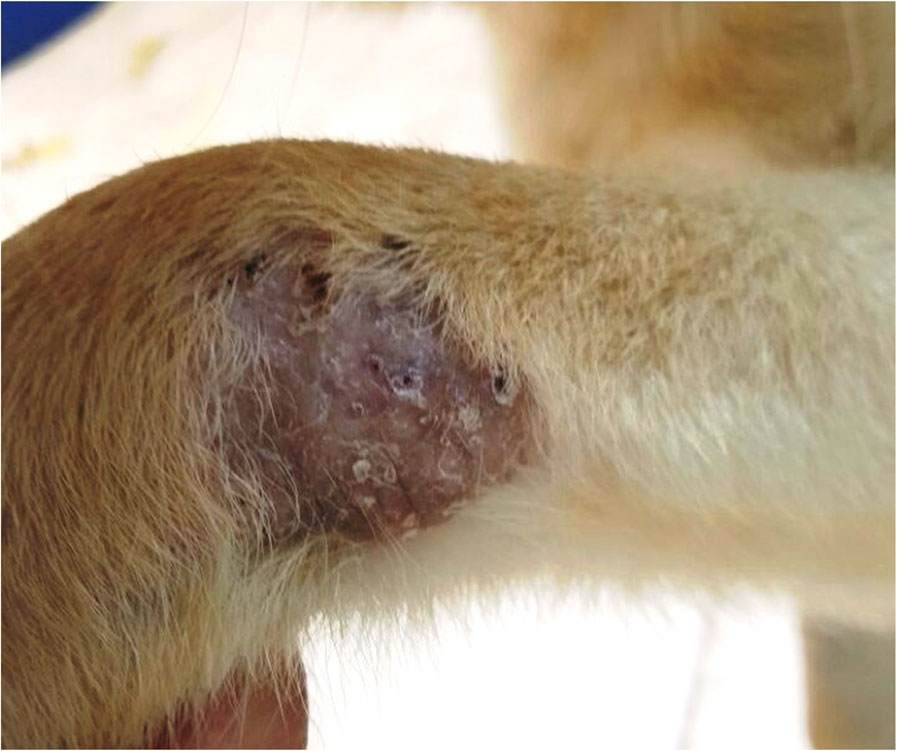
Cutaneous skin lesion over the carpus in dog case no. 1 infected with *Leishmania major*. Image taken at 6 months of age prior to treatment

A 5-month-old mixed breed dog male castrated dog that had been adopted from an animal shelter in Jerusalem, Israel, was admitted to a veterinary clinic with lethargy, anorexia and dermatologic abnormalities. The dog had suffered from skin lesions on its face and legs when it was brought to the shelter 3 weeks earlier. The skin lesions consisted of unilateral periocular alopecia with ulceration, scales and serous discharge around the right eye (Fig. 1a), ulceration in the left hind foot pad and ulcerative scaly lesions on the left front leg over the carpus (Fig. 2) and left hind leg over the tarsus. Physical examination at the veterinary clinic was otherwise normal. A complete blood count (CBC) indicated mild normocytic normochromic anemia with a packed cell volume (PCV) of 31% (reference range 32–55%) and serum biochemistry panel was within normal limits. Skin scrapings from the lesions were negative for *Demodex* spp., and culture for dermatophytes was also negative. ELISA serology for *L. infantum* submitted to the HUSVM was negative, however, a skin biopsy taken from the eye lesion and submitted for PCR for *Leishmania* spp. at the HUSVM and was positive for *Leishmania* by HRM-PCR with a melt curve pattern compatible with *L. major* which was confirmed by sequencing of a 240 bp DNA PCR product that had 100% identity to *L. major* (GenBank: KP773413.1) and was deposited in GenBank (KY524299). The dog was referred to the HUSVM teaching hospital for further diagnosis and follow up. It was admitted a month after its initial visit to the referring veterinarian and on physical examination skin lesions on the feet were in a similar state to that found a month earlier but the periocular lesion appeared to improve with the disappearance of the serous discharge and some regrowth of hair (Fig. 1b). Complete blood count (CBC) showed worsening of the normoncytic normochromic anemia with a PCV of 25%. Serum biochemistry and urinalysis were within normal limits. Left and right conjunctival swabs, prescapular lymph node aspirates and urine were negative for *Leishmania* by kDNA PCR [8]; however blood was positive by the same PCR protocol and confirmed by DNA sequencing with the closest identity to *L. major* (GenBank: EU370907.1). ELISA serology for *L. infantum* was negative again. Treatment with 10 mg/kg of allopurinol orally twice daily was started and the owners were instructed to apply a topical insecticide against sand fly bites on the dog. The dog was rechecked at the HUSVM teaching hospital 7 weeks after the beginning of treatment. The dog’s skin condition had improved and the periocular lesion had almost disappeared under dense regrowth of hair covering moderately thickened small skin scars (Fig. 1c). The skin lesions over the left tarsus and carpus have also healed and were almost unapparent. The dog was no longer anemic with a PCV of 39% and serum biochemistry was within normal limits. PCR of the blood was negative and serology was repeatedly negative. The owners were instructed to continue allopurinol treatment and recheck the dogs 3 months later.

#### Case no. 2

A 3-month-old stray female intact pup was admitted to the HUSVM teaching hospital from Tel Aviv in Israel with a complaint of lethargy. On physical examination the dog was thin with a decreased body condition score (2/9), a normal rectal body temperature and with mild serous ocular discharge. On CBC the dog was anemic (PCV 22%) with a regenerative anemia, and moderately thrombocytopenic (110 × 10^9^ thrombocytes/l; reference 150–400 × 10^9^/l). Stained blood smear evaluation revealed inclusions in thrombocytes suspected as *Anaplasma platys* morulae and large form *Babesia* sp. organisms in erythrocytes. PCR for *Anaplasma* sp. and for *Babesia* sp. was carried out using the piroplasmid and EC-16S primers for *Babesia* and *Anaplasma*/*Ehrlichia* spp., respectively, as previously described [12, 13]. Both PCRs were positive and following DNA sequencing it was confirmed that the dog was infected with *Babesia vogeli* (99% identical to GenBank HQ662635.1) and *A. platys* (99% identical to GenBank JQ976643.1). No serum was available from this dog for serology. The dog was treated with doxycycline at 10 mg/kg P.O. daily for 14 days against anaplasmosis, and imidocarb dipropionate at a reduced dose of 2.5 mg/kg due to the dog's young age by single I.M. injection against the babesial infection with premedication of atropine at 0.05 mg/kg I.M. to prevent the possible cholinergic effects of imidocarb dipropionate. In addition, the dog was treated with an antibiotic eye ointment containing 5% tetracycline.

The dog was hospitalized, received I.V. fluid transfusion and followed up clinically. It was discharged after 4 days when it improved clinically and regained normal activity and was adopted by new owners that were instructed to come for a recheck and repeated imidocarb dipropionate injection after 10 days but did not return for medical treatment and refused further contact.

Pre-treatment blood from the dog was submitted to the laboratory to attempt isolation of *A. platys*, which has not been grown successfully in culture to date. It was initially transferred to a temporary medium, which has a similar composition to the medium used as a basis for culture of *Leishmania*, before seeding in a cell culture which was not carried out eventually as *Leishmania* was detected in the transfer medium. Essentially, 100 μl of blood was incubated in an equal volume of minimum essential medium (MEM) (Sigma, Saint Louis, USA)/Leibovitz's L15 medium (Gibco, Carlsbad, CA, USA), supplemented with 20% fetal calf serum (Biological industries, Beit Haemek, Israel), 10% tryptose phosphate broth solution (TPB, Sigma-Aldrich, Saint Louis, USA), 1% penicillin-streptomycin (Sigma-Aldrich, Saint Louis, USA) and 1% L-glutamine (Biological industries, Beit Haemek, Israel). After 2 h, medium was removed and replaced by fresh medium and incubated at 29 °C. When the medium was tested by microscopy after 7 days, it contained propagating organisms which appeared similar in shape to *Leishmania* spp. promastigotes and indeed were identified as *L. tropica* by HRM-PCR and DNA sequencing (100% identical to GenBank GU561643.1). This *L. tropica* sequence was deposited in GenBank (KY524300). To verify *Leishmania* spp. infection in the dog, blood kept from the dog's CBC was tested by PCR for *Leishmania* and was also positive by kDNA PCR [8]. This diagnosis was surprising since the dog did not show clinical signs that were suspicious of leishmaniosis such as skin lesions.

### Comparative serology

**Fig. 3:**
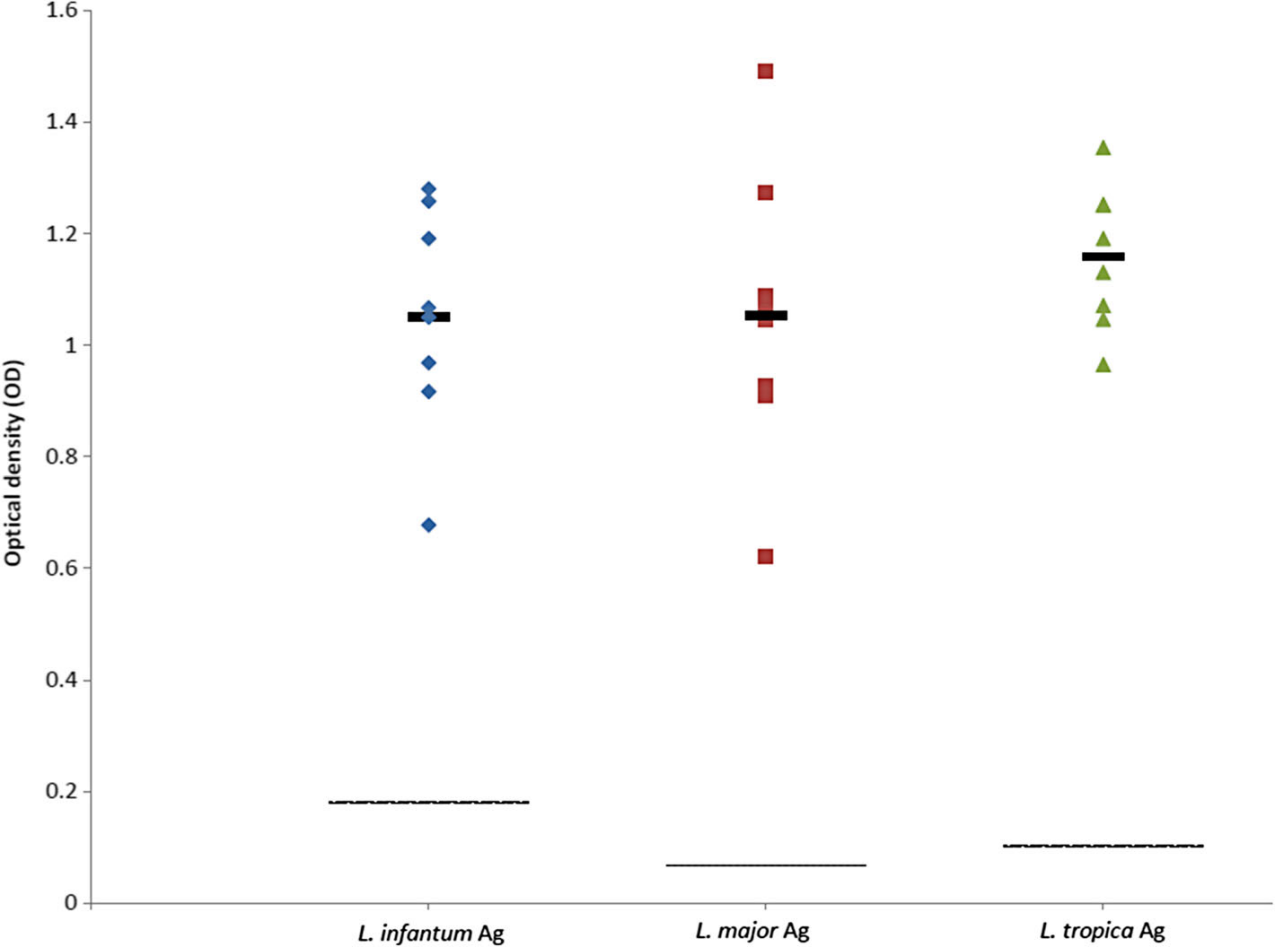
Scatter plot showing reactivity of sera tested by ELISA from eight dogs infected with *Leishmania infantum*. Seroreactivity was tested with antigens of *L. infantum*, *L. major* and *L. tropica*. Y-axis shows calibrated optical density (OD). The thick black line represents the mean value. The intermittent line represents the serological cut-off value. No significant differences were found between OD readings of the same sera with the different antigens (*F*_(2,23)_ = 0.72, *P* = 0.498)

**Fig. 4:**
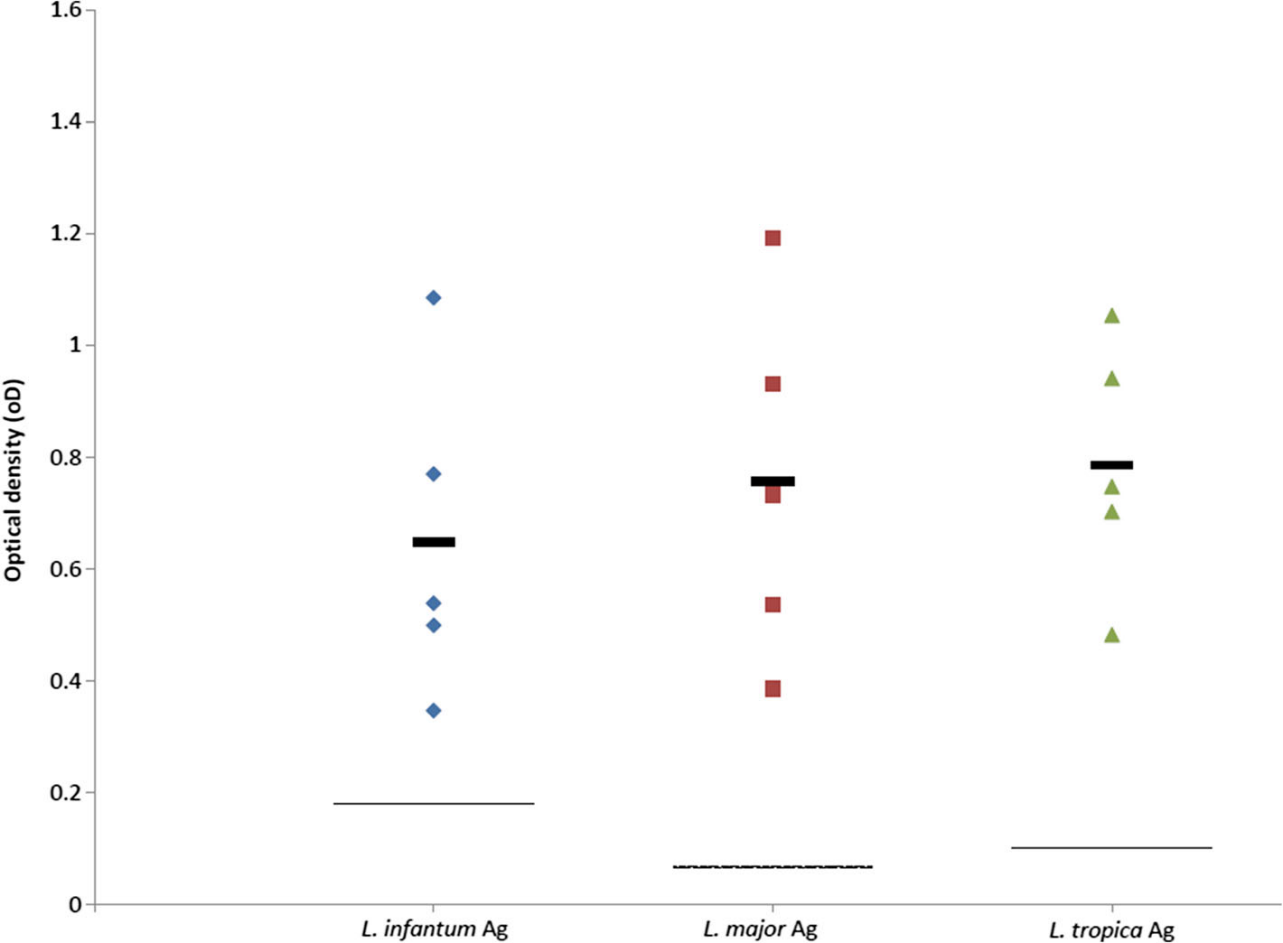
Scatter plot showing reactivity of five sera tested by ELISA from two dogs infected with *Leishmania tropica*. Seroreactivity was tested with antigens of *L. infantum*, *L. major* and *L. tropica*. Y-axis shows calibrated optical density (OD). The thick black line represents the mean value. The intermittent line represents the serological cut-off value. No significant differences were found between OD readings of the same sera with the different antigens (*F*_(2,14)_ = 0.336, *P* = 0.721)

**Fig. 5:**
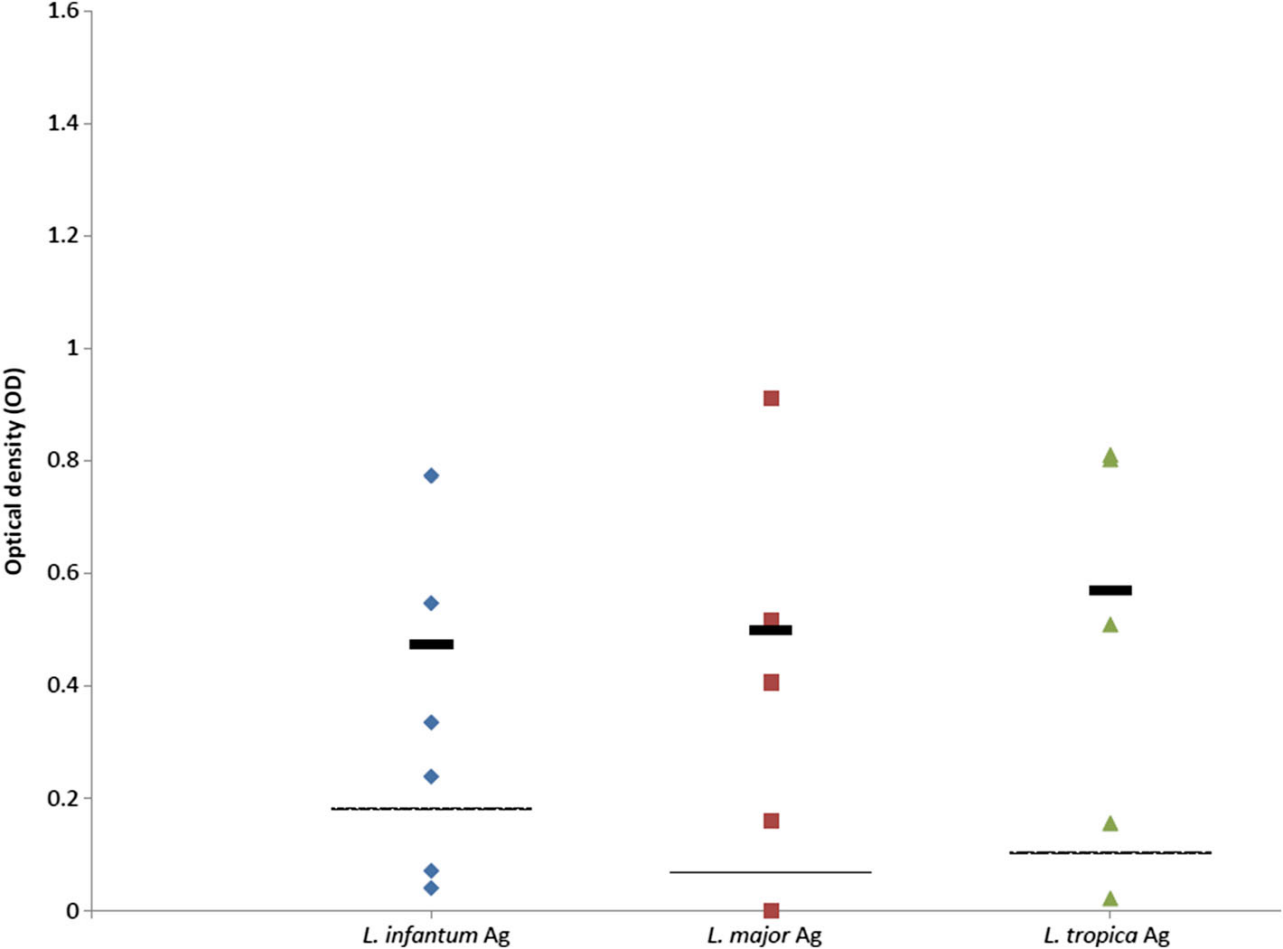
Scatter plot showing reactivity of six sera tested by ELISA from two dogs infected with *Leishmania major*. Seroreactivity was tested with antigens of *L. infantum*, *L. major* and *L. tropica*. Y-axis shows calibrated optical density (OD). The thick black line represents the mean value. The intermittent line represents the serological cut-off value. Samples 6835 and 0026 were omitted from the mean calculation due to dog case no. 2 being seronegative. No significant differences were found between OD readings of the same sera with the different antigens (*F*_(2,11)_ = 0.118, *P* = 0.824)

Altogether 19 sera samples from 11 dogs infected with one of the three *Leishmania* spp. studied were available: eight from individual dogs with *L. infantum*; six from two dogs with *L. major* infection (four from one dog taken over a five months period and two from a second dog taken over three months); and five from a dog with *L. tropica* infection taken at different time points over 26 months (see Additional file 1: Table S1). The serological cut-off values calculated from seronegative dogs were 0.18, 0.068 and 0.101 for *L. infantum*, *L. major* and *L. tropica*, respectively. Sera from all dogs reacted positively with all three *Leishmania* species antigen except for the two samples from case no. 2 with *L. major* infection described in this study which did not react with *L. major* antigen nor with the two other leishmanial species antigens (Additional file 1: Table S1). After omitting dog no. 2’s negative results, no significant differences (*F*_(2,23)_ = 0.72, *P* = 0.498 for *L. infantum* antigen; *F*_(2,11)_ = 0.118, *P* = 0.890 for *L. major* antigen; *F*_(2,14)_ = 0.336, *P* = 0.721 for *L. tropica* antigen) were found between reactivity of dog sera to the antigen of the infecting species as expressed in OD, and reactivity to the other two *Leishmania* species antigens (Figs. 3, 4 and 5).

Sera of dogs infected with *L. infantum* and *L. tropica* reacted positively with the rK39 antigen kit, while the sera of dogs with *L. major* infection were negative by this antigen kit.

## Discussion

This study describes unique cases of dog infection with *L. major* and *L. tropica*, two common agents of cutaneous leishmaniosis in people in the Middle East and North Africa, albeit rarely reported as associated with disease in dogs [1, 3]. These cases provide additional clinical, diagnostic and therapeutic information on these infections in dogs and add to other reports based on molecular or enzymatic biochemical characterization of clinical canine *L. major* [7, 14–16] and *L. tropica* infections [5, 6, 17–19]. In addition, they provided an opportunity to compare the reactivity of serum from dogs to different leishmanial antigens and evaluate the value of serological testing in canine infection with *L. major* and *L. tropica*.

The dogs described in this study and other molecularly characterized reports of *L. major* and *L. tropica* canine infections [6, 7, 19] have described animals younger than 1-year-old infected with *L. major* [7] and both young [6, 19] and older dogs above 5 years of age infected with *L. tropica* [5]. Skin lesions in *L. major* infected dogs from this study and a previous report [7] were ulcerative and located on the muzzle, feet and foot pads and not associated with generalized lymph node enlargement and palpable splenomegaly. In contrast, in *L. tropica* infection, skin lesions were mucocutaneous and proliferative in two cases of young dogs [6, 19], or associated with pustular dermatitis, lymphadenomegaly and splenomegaly [5]. Older descriptions of *L. tropica*-infected dogs diagnosed based on culture and enzymatic characterization describe dermatitis with facial papules and no other clinical manifestations in seven dogs from Morocco [20] or a severe disease, with poor body condition, skin and internal organ pathology similar to canine viscerocutaneous leishmaniosis caused by *L. infantum* [17, 18].

The descriptions of only skin lesions in canine *L. major* infection while canine *L. tropica* infection may manifest as a skin disease which may in some cases also disseminate to visceral organs and cause generalized disease, are in agreement with these diseases in people where *L. major* causes cutaneous disease but *L. tropica* is also involved with human visceral leishmaniosis [21, 22].

The hematological and serum biochemistry findings in canine *L. major* infection described here (case no. 1) indicated a mild anemia with no serum biochemistry abnormalities. A previous report of *L. major* infection [7] described no CBC and serum chemistry abnormalities. The laboratory findings for the *L. tropica* infected dog (case no. 2) with anemia, leukopenia and mild thrombocytopenia were probably affected by the infections with *B. vogeli* and *A. platys* and should not be interpreted as necessarily associated with *L. tropica* infection. A previous case of *L. tropica* had a mild leukocytosis and eosinophilia with no anemia [6], while an Iranian young dog with *L. tropica* infection had a reported normal hemogram [19].

Overall, the hematology and serum biochemistry findings from *L. major* and *L. tropica* infected dogs suggest that they present a different picture from the typical findings of hyperglobulinemia, hypoalbuminemia and anemia found in dogs with generalized canine leishmaniosis caused by *L. infantum* [23]. This may be due to the visceralization of *L. infantum* with changes in internal organs associated with this chronic infection.

Detection of *L. major* DNA by PCR in blood was successful in the dog reported here and negative on multiple dates in a previous report [7] where the prescapular lymph node was positive. In *L. tropica* infected dogs, blood PCR was positive in the dog reported here and parasite culture was grown out of the dog's blood; however, blood PCR was negative in a previous report on a dog with mucocutaneous leishmaniosis [6]. Therefore, it can be concluded blood PCR may be positive in canine *L. major* and *L. tropica* infections; however, it is probably not a sufficiently sensitive and reliable test for confirming these infections. Blood PCR is also not an optimal test for confirming canine *L. infantum* infection and bone marrow or lymph node PCR are preferred [23].

ELISA serology with crude promastigote antigen was not found to be distinctive between *Leishmania* species in dogs in this study as there was no significant association between the leishmanial species infecting the dogs and their seroreactivity to its antigen when compared to antigen of other species. Furthermore, the dog with *L. major* infection (case no. 1) was seronegative for all antigens in contrast to a dog with *L. major* infection described in a previous study [7] whose serum was reactive with the antigens of all *Leishmania* spp. in this study.

Canine serological cross-reactivity between *Leishmania* spp. antigens has been described before and dogs infected with *L. infantum* (syn. *Leishmania chagasi*) from Brazil have been shown to respond to antigen from *L. major*-like antigen by ELISA [24] and also to *L. braziliensis* antigen [25]. Furthermore, sera from Brazilian *L. infantum* (*chagasi*)-infected dogs was reactive by western immunoblotting with several antigens from whole promastigote antigens of *Leishmania guyanensis*, *Leishmania amazonensis* and *L. braziliensis* [26]. Therefore, cross-reactivity amongst the Old World species of *L. major*, *L. tropica* and *L. infantum* as found in dogs in the current study is not surprising, and it is important to note that ELISA has not been found distinctive for the infecting *Leishmania* species with regard to the OD level of reactivity in this study.

The use of recombinant antigens such as the rK39 for serology is an additional diagnostic tool. The rK39 is a kinesin-like protein derived from *L. infantum* (*chagasi*) which contains a 39 amino acid repeat conserved in *L. infantum* and the closely related *L. donovani* [27]. Serology testing to rK39 is available in qualitative dipsticks and also in quantitative ELISA assays. In this study, sera from *L. infantum-* and *L. tropica-*infected dogs was positive with the rK39 dipstick, but not sera from *L. major-*infected dogs. Positive response to the rK39 dipstick has also been reported from an Iranian young dog with mucocutaneous lesions due to *L. tropica* [19] suggesting that this test might be useful for the detection of canine *L. tropica* infection. The fact that sera from dogs with *L. major* infection did not react with the rK39 dipstick is interesting but needs further testing with more dogs because although one dog responded to all the crude antigen ELISAs of the three *Leishmania* spp. but not to rK39, the other dog was not reactive with these three antigens. The absence of a detectable serologic response to *L. major* in one of the dogs can be explained by the fact that this species causes apparently cutaneous-restricted infection in humans and possibly also in dogs, and does not elicit a detectable serologic response in some infected humans [28].

The dog with *L. major* infection (case no. 1) in this study responded well to allopurinol treatment at 10 mg/kg twice daily, and its skin lesions improved and almost disappeared within 7 weeks of treatment. This successful response to allopurinol treatment is similar to that described for a previous canine *L. major* case treated with allopurinol and described by us [7]. The response of canine *L. tropica* to the same treatment regimen was also successful in a previously described case [6] and unfortunately the dog in the current study (case no. 2) was not followed up due to loss of contact with its owners. It should be noted that dogs with *L. major* and *L. tropica* infections in the comparative serology study were also treated with allopurinol and this may have affected their serological responses in samples taken after the beginning of treatment by decreasing the amount of antibodies; however, it can be presumed that this would affect the response to different *Leishmania* spp. antigens similarly.

Although current understanding of the clinical and diagnostic findings and treatment of canine *L. major* and *L. tropica* infection is limited by the small number of dogs diagnosed and treated for these infections, this study together with previous reports add to the characterization of disease associated with these two dermatropic species in dogs and to knowledge about its management.

## Conclusions

This study further elucidates the clinical and parasitological findings in canine *L. major* and *L. tropica* infections. It suggests that ELISA serology with whole promastigote antigen is not distinctive between *L. infantum*, *L. major* and *L. tropica* canine infections and in some cases clinical *L. major* infections may not be seropositive, and it indicates that PCR with DNA sequencing from the affected tissues or blood should be used in dogs to discriminate between infections with these three Old World *Leishmania* spp.

## Additional file

Additional file 1: Table S1. Optical density readings (OD) of sera from dogs naturally infected with *Leishmania infantum*, *L. major* or *L. tropica* tested by ELISA for reactivity with crude promastigote antigen of these three species. (DOCX 17 kb)

## Abbreviations

CBC: Complete blood count
ELISA: Enzyme-linked immunosorbent assay
HRM: High resolutions melt
HUSVM: Hebrew University School of Veterinary Medicine
ITS: Internal transcribed spacer
OD: Optical density
PCR: Polymerase chain reaction
