# Supplementary material for: Canine leishmaniosis caused by Leishmania major and Leishmania tropica: comparative findings and serology
Source: Parasit Vectors. 2017 Mar 13;10:113. doi: 10.1186/s13071-017-2050-7 (PMC5346844; doi:10.1186/s13071-017-2050-7)
Supplement: Additional file 1: Table S1. — Optical density readings (OD) of sera from dogs naturally infected with Leishmania infantum, L. major or L. tropica tested by ELISA for reactivity with crude promastigote antigen of these three species. (DOCX 17 kb) [file 13071_2017_2050_MOESM1_ESM.docx]

**Additional file 1. Table S1** Optical density readings (OD) of sera from dogs naturally infected with *Leishmania infantum*, *L. major* or *L. tropica* tested by ELISA for reactivity with crude promastigote antigen of these three species.

| **Infecting *Leishmania* sp.** | **Sample number** | **Dog number** | ***L. infantum* antigen** | ***L. major* antigen** | ***L. tropica* antigen** |
| --- | --- | --- | --- | --- | --- |
| *L. infantum* | 8710 | 1 | 0.917 | 1.047 | 0.965 |
| *L. infantum* | 8714 | 2 | 1.068 | 0.910 | 1.252 |
| *L. infantum* | 8793 | 3 | 0.969 | 1.072 | 1.191 |
| *L. infantum* | 8757 | 4 | 1.258 | 1.274 | 1.354 |
| *L. infantum* | 8799 | 5 | 1.191 | 1.492 | 1.130 |
| *L. infantum* | 8818 | 6 | 1.280 | 0.928 | 1.252 |
| *L. infantum* | 8848 | 7 | 1.050 | 0.622 | 1.252 |
| *L. infantum* | 8632 | 8 | 0.678 | 1.089 | 1.072 |
| *L. major* | 8647 | 9 | 0.546 | 0.516 | 0.802 |
| *L. major* | 8649 | 9 | 0.773 | 0.911 | 0.810 |
| *L. major* | 4006 | 9 | 0.335 | 0.406 | 0.508 |
| *L. major* | 5052 | 9 | 0.238 | 0.159 | 0.155 |
| *L. major* | 6835 | 10 (case2) | 0.071 | 0.000 | 0.021 |
| *L. major* | 0026 | 10 (case 2) | 0.04 | 0.000 | 0.000 |
| *L. tropica* | 9910 | 11 | 0.500 | 0.733 | 0.702 |
| *L. tropica* | 4939 | 11 | 1.085 | 1.191 | 1.053 |
| *L. tropica* | 1064 | 11 | 0.539 | 0.537 | 0.748 |
| *L. tropica* | 4030 | 11 | 0.347 | 0.386 | 0.483 |
| *L. tropica* | 7489 | 11 | 0.770 | 0.932 | 0.941 |
